# Single molecule mRNA fluorescent *in situ* hybridization combined with immunofluorescence in *S. cerevisiae:* Dataset and quantification

**DOI:** 10.1016/j.dib.2020.105511

**Published:** 2020-04-11

**Authors:** Anna Maekiniemi, Robert H. Singer, Evelina Tutucci

**Affiliations:** a Department of Anatomy and Structural Biology, Albert Einstein College of Medicine, Bronx, NY 10461, United States; b Gruss-Lipper Biophotonics Center, Albert Einstein College of Medicine, Bronx, NY 10461, United States; c Janelia Research Campus of the HHMI, Ashburn, Virginia 20147, United States; d Systems Biology Lab, Amsterdam Institute of Molecular and Life Sciences (AIMMS), Vrije Universiteit Amsterdam, Amsterdam, the Netherlands

**Keywords:** Single molecule mRNA FISH, smFISH, Immunofluorescence, smFISH-IF, Single cell, Imaging analysis, Gene expression profiling, Gene expression

## Abstract

Single-molecule fluorescent in situ hybridization (smFISH) has emerged as a powerful technique that allows one to localize and quantify the absolute number of mRNAs in single cells. In combination with immunofluorescence (IF), smFISH can be used to correlate the expression of an mRNA and a protein of interest in single cells. Here, we provide and quantify an smFISH-IF dataset in *S. cerevisiae.* We measured the expression of the cell cycle-controlled mRNA *CLN2* and the cell cycle marker alpha-tubulin. The smFISH-IF protocol describing the dataset generation is published in the accompanying article “Simultaneous detection of mRNA and protein in *S. cerevisiae* by single-molecule FISH and Immunofluorescence” [1]. Here, we analyze the smFISH data using the freely available software FISH-quant [2]. The provided datasets are intended to assist scientists interested in setting up smFISH-IF protocol in their laboratory. Furthermore, scientists interested in the generation of imaging analysis tools for single-cell approaches may find the provided dataset useful. To this end, we provide the differential interference contrast (DIC) channel, as well as multicolor, raw Z-stacks for smFISH, IF and DAPI.

Specifications Table

**Table:** 

Subject	Biology, Cell Biology, Biophysics
Specific subject area	Gene expression analysis, Single molecule mRNA imaging, Fluorescence imaging analysis
Type of data	Table Image Graph Figure Analysis files
How data were acquired	Data were acquired using a wide-field fluorescence microscope coupled to a CCD camera, a motorized stage driven by ultrasonic piezo technology for precise Z-positioning and a 120 W mercury short arc lamp as fluorescence illumination system. Model and make of the instruments used: Olympus BX-63 epifluorescence microscope equipped with Ultrasonic stage and UPlanApo 100 ×, 1.35NA oil-immersion objective (Olympus). An X-Cite 120 PC Lamp (EXFO), an ORCA-R2 Digital Interline CCD Camera (C10600-10B; Hamamatsu; 6.45 µm-pixel size) mounted using U-CMT and 1X-TVAD Olympus c-Mount Adapters, and zero-pixel shift filter sets: DAPI-5060C-Zero, FITC-5050-000, Cy3-4040C-Zero, and Cy5-4040C-Zero from Semrock. Software used for instrument control and image acquisition: MetaMorph (Molecular Devices).
Data format	Raw: TIFF Analyzed: Matlab files Filtered: TIFF CellProfiler pipeline
Parameters for data collection	The data were collected in the commonly used auxotrophic *S. cerevisiae* strain BY4741. Cells were grown at 26 °C in Synthetic Complete medium (SC) with 2% glucose. Cells grown to exponential phase were fixed to perform smFISH-IF.
Description of data collection	smFISH-IF data were collected by using a wide-field fluorescence microscope. A monolayer of yeast cells attached on a glass coverslips were imaged at different wavelengths to detect the following fluorophores: Alexa 647 or Alexa 488 (IF); Quasar 570 (smFISH); DAPI (nuclear staining); DIC (cell outline). Ten to twenty different stage positions were acquired per experiment (to reach 1000 cells for quantification). At each stage position, and for each fluorescent channel, we collected 41 Z-stacks every 200 nm. For DIC, one single Z-stack was acquired at the focal plane.
Data source location	Institution: Department of Anatomy and Structural Biology, Albert Einstein College of Medicine City/Town/Region: Bronx, NY Country: USA Institution: Systems Biology Lab, Vrije Universiteit Amsterdam City/Town/Region: Amsterdam Country: The Netherlands
Data accessibility	Repository name: [Mendeley] Data identification number: [DOI: 10.17632/bcmn9cxyzs.4] Direct URL to data: [https://data.mendeley.com/datasets/bcmn9cxyzs/4]
Related research article	Authors Tutucci Evelina and Robert H. Singer Title “Simultaneous detection of mRNA and protein in *S. cerevisiae* by single molecule FISH and Immunofluorescence” Journal Methods in molecular Biology DOI https://doi.org/10.1007/978-1-0716-0712-1, ISSN: 1064–3745.

## Value of the data

• smFISH-IF data are useful to correlate the expression of an mRNA of interest to the expression of a protein in single cells. This approach has the potential to reveal the subcellular localization of mRNAs, the cell-to-cell variability in gene expression, and to precisely quantify the absolute number of mRNAs in the cytoplasm and at the transcription site.
• Scientists interested in setting up the smFISH-IF protocol in their lab could use this dataset as a reference to compare their dataset to an established protocol.
• The presented data were collected using *S. cerevisiae* as a model system. We believe that the approach is relevant to other organisms. Here, we provide an overview of the smFISH-IF protocol and imaging analysis process by making available the raw data and the smFISH quantification performed with the software FISH-quant [2].
• The increasing use of microscopy in life sciences is rising the need for imaging analysis tools. Further automation of the quantification process (e.g. by using machine learning algorithms) will expand the application of life science microscopy data. Thus, software developers involved in the generation of imaging analysis tools for gene expression analysis and cell physiology may find useful the provided dataset.

## 1 Data

**Fig. 1:**
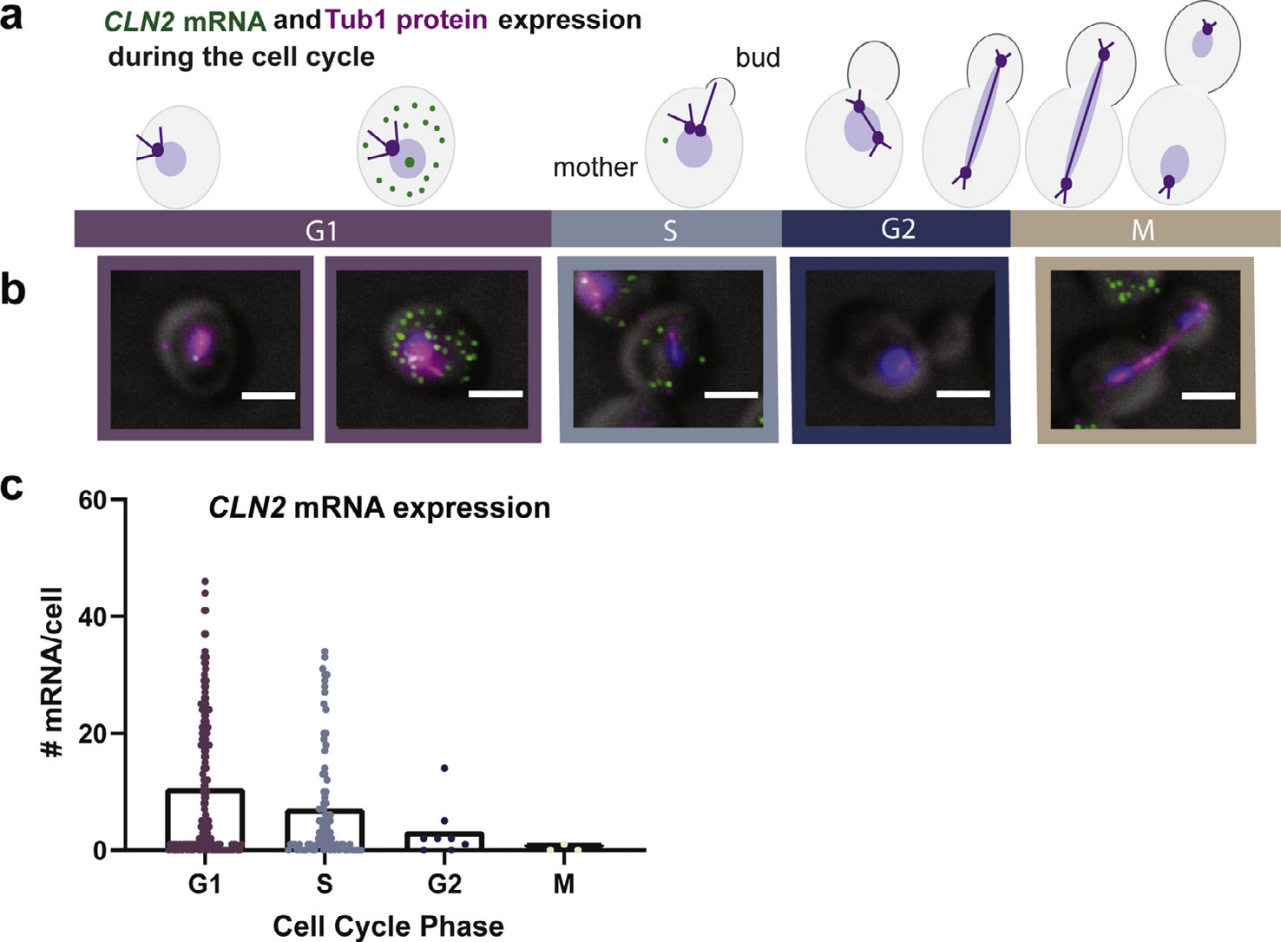
smFISH-IF for the cell-cycle mRNA *CLN2* and the protein tubulin 1. (a) Schematic of *CLN2* mRNA expression during the cell cycle. Green dots represent *CLN2* mRNA in the cytoplasm and transcription sites (TS) in the nucleus. TS are identified by the higher brightness compared to the single cytoplasmic mRNAs. Tubulin (purple) co-localizes with the spindle pole body which is duplicated during S phase. The bud emergence starts during S phase and ends with the formation of the daughter cell. During anaphase, the microtubules stretch between the mother and the daughter cell [11]. The *CLN2* mRNA is transcribed during late G1 and is homogeneously dispersed in the cytoplasm. (b) MERGE Maximally projected images: *CLN2* mRNA smFISH Quasar 570 (green), anti-tubulin IF Alexa 488 (magenta) and DAPI (blue) merged to a single plane DIC image (gray). The smFISH Quasar 570 (green) channel has been filtered using FISH quant. Scale bars = 2 µm. (c) Quantification of the number of mRNAs per cell in each cell cycle phase. (For interpretation of the references to color in this figure legend, the reader is referred to the web version of this article.)

smFISH has emerged as a powerful technique that allows one to localize and quantify the absolute number of mRNAs in single cells [3]. In combination with immunofluorescence, smFISH can now be used to correlate the expression of an mRNA of interest to that of a protein in single cells [4], [5], [6]. Because the quantifications are strictly dependent on the quality of the primary data, it is important to achieve high-quality smFISH datasets. Here, we provide three smFISH-IF datasets that we deposited in the public repository http://dx.doi.org/10.17632/bcmn9cxyzs.4 [7]. The smFISH was done to detect the cyclin 2 mRNA (*CLN2*), which is a cell cycle-regulated mRNA periodically expressed during the G1 phase [8]. The immunofluorescence was done to detect the protein tubulin 1 that was used to monitor the cell cycle phase (Fig. 1a).

### 1.1 Raw images

Three independent biological replicates were deposited: EXP1_BY4741_smFISH-IF_Tub1_CLN2, EXP2_BY4741_smFISH-IF_Tub1_CLN2 and EXP3_BY4741_smFISH-IF_Tub1_CLN2.

For each experiment, we provide multiple stage positions using the following filter sets: a) CY5 or FITC: to detect the IF, revealed using a secondary antibody conjugated to the fluorophore Alexa647 or Alexa488, respectively. b) CY3: to detect the smFISH, revealed using probes conjugated with the fluorophore Quasar 570. c) DAPI: to detect the nuclear staining performed using DAPI. d) DIC: differential interference contrast image. For each stage position and wavelength, we collected 41 z-stacks every 200 nm in the following order: CY5 or FITC, CY3 and DAPI channels. The files are saved in TIFF format.

Representative smFISH-IF results are shown in Fig. 1b, whereby the different fluorescent channels are merged to the DIC channel. The different fluorescent channel images are the result of maximal projections of the Z-stacks where the cells are located (usually 20–25 Z-planes), generated using Image J [9]. The CY3 channel has been filtered using the Gaussian filter in FISH-quant. The DIC images have a systematic drift compared to the fluorescence images due to our microscope characteristics. This drift is corrected using the x, y “translate” function in Fiji.

The three datasets are representative of typical smFISH-IF results. Datasets 1 and 2 have a few imperfections that can be found in this type of experimental setting and are discussed in the following paragraphs.

In dataset 1, positions 6 and 7 show a significant drift in the *Z* directions (about 1 pixel per Z-plane). This problem can be detected using, for instance, the Fiji function: *Image>Stacks>Orthogonal views*, and it can be caused by a small bubble in the oil or if the objective is not clean. A small bubble is likely the cause for positions 6 and 7 since the positions of EXP1 were collected consecutively, but only at these two positions, we found the drift. These images can still be used to quantify the number of mRNAs per cell using FISH-quant (see output results). The filtered images still allow the identification of mRNA spots. However, if one requires to localize mRNAs with high precision (e.g. for co-localization experiments), we recommend discarding these types of images.

In dataset 2, we observe some debris in the IF channel (CY5) at the bottom of the slide. This can happen if the excess of fluorescently labeled secondary antibodies used for IF detection is not completely removed during the washes. This problem can be solved by increasing the number and the stringency of washes during the IF protocol, or by increasing the concentration of bovine serum albumin (BSA) used to block the non-specific binding of the antibodies. In the case of tubulin, used in this experiment as a cell cycle marker, we believe the data can still be used for cell cycle scoring based on the reasons described in the following paragraph.

### 1.2 Cell cycle phase scoring of cells

The cell cycle phase of each cell was determined using the morphology of the cell and the tubulin IF signal intensity. The software Cellprofiler was used to quantify the integrated intensity of the IF signal within the cell outlines and for the maximal projection of the Z-sections containing the cells, excluding the bottom of the slide. Thus, most of the debris is not quantified. This allows one to distinguish cells in the G1 phase from cells in the following phases of the cell cycle (S to M phase) due to an increase in the tubulin signal that is caused by a spindle duplication during S phase [10]. The CellProfiler pipeline used in this study was deposited in the public repository http://dx.doi.org/10.17632/bcmn9cxyzs.4.

After quantifying the tubulin signal, we combine these measurements with the morphology of the tubulin and cell (based on DIC) to manually score the cells according to their cell cycle phase. This scoring is done in Fiji, using the plugin Cell Counter [9]. G1 phase cells can be identified by the single, nuclear localized, spindle pole body and round morphology of the cells as well as a lower tubulin intensity. The emergence of a small bud identifies S-phase cells. G2 phase cells can be identified by their larger bud and duplicated spindle pole body with tubulin stretching into the emerging bud. M-phase cells can be identified by the extended spindle and dividing nucleus (Fig. 1a). A CSV file is created from Fiji, containing the information on the position and scoring group of each cell. This file can then be imported into the outline designer in FISH-quant using the “Load Groups” menu option. After the FISH-quant analysis, described in detail below, one has the possibility of sorting the mRNA expression results according to the cell cycle group (Fig. 1c).

### 1.3 Cell outlines

Together with the raw images, we deposited the smFISH analysis files that were generated using FISH-quant [2] (see Experimental Design, Materials, and Methods for more details). The outlines for the ten stage positions were generated using Cellprofiler (the pipeline is provided with V3 of FISH-quant) and imported using the built-in FISH-quant option.

**Fig. 2:**
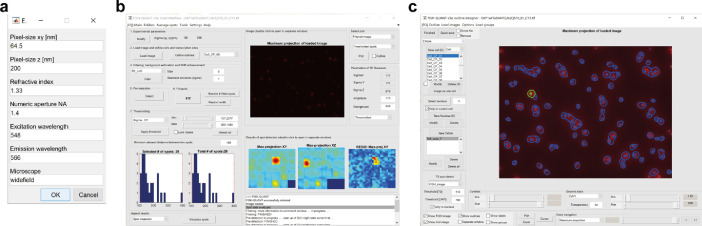
Workflow of the single mRNA molecule averaging using FISH-quant. (a) Microscope parameters for single mRNA detection. (b) FISH-quant main interface showing the parameters used for background estimation and signal to noise ratio (SNR) enhancement. (c) FISH-quant outline designer interface showing the cell outlines generated using Cellprofiler merged on the smFISH image.

The text files (e.g., EXP1wtTub647CLN2Q570_01_CY3__outline) describe the coordinates of nuclear and cell outlines for a total of 1018 cells. The files are imported in FISH-quant main interface (Fig. 2a) after loading the corresponding raw smFISH stage position (41 Z-stacks) and loading the corresponding outline file (Fig. 2b). At this stage, the quality of the outlines is verified and corrected if necessary. Furthermore, at this stage, transcription sites (TS) auto-detection settings are defined (e.g. threshold for TS brightness, and DAPI brightness).

### 1.4 FISH-quant batch analysis files

Once all cell outlines and transcription sites are defined, we set the parameters of single mRNA detection. Using FISH-quant main interface, the image is filtered using the parameters shown in Fig. 2a. A single cell expressing the mRNA of interest, is chosen to set the initial parameters using the pre-detection tools. Once the parameters best identifying the mRNAs in the chosen cell are found, they are applied to the entire image and then saved as a text file. We deposited the file describing the mature mRNA settings used to analyze EXP1 (EXP1wtTub647CLN2Q570_01_CY3__settings_MATURE).

**Fig. 3:**
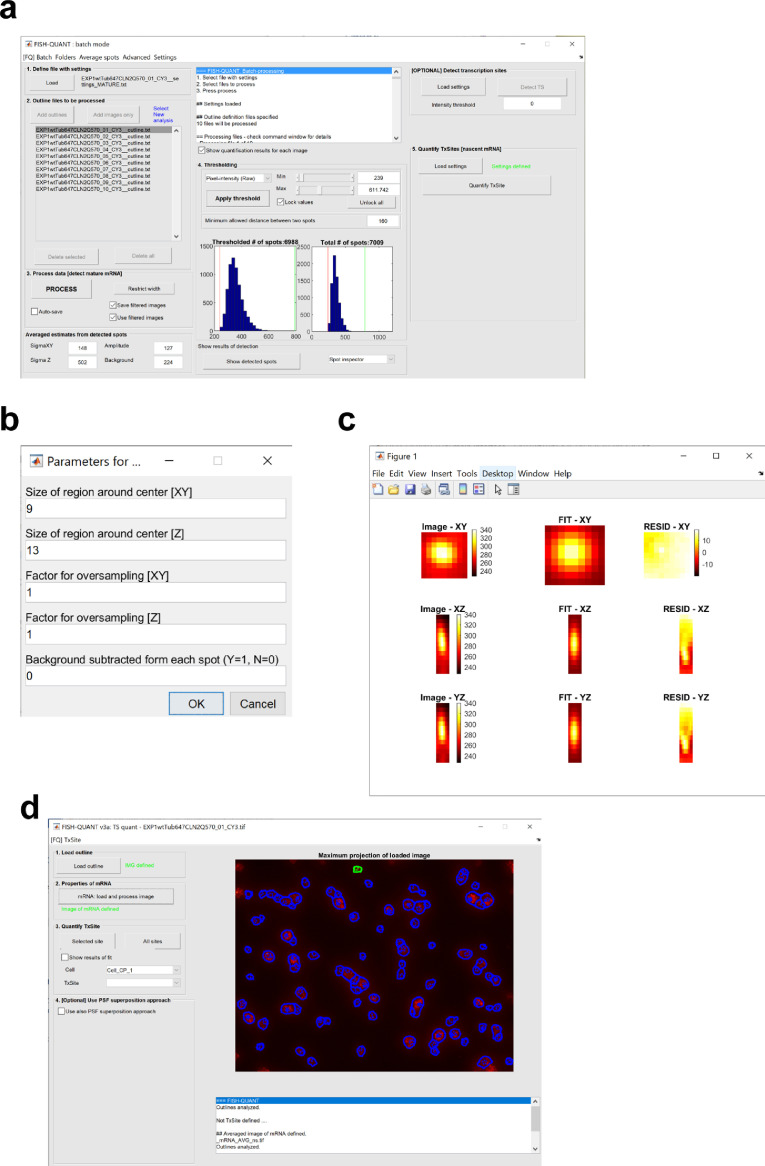
Workflow of the single mRNA molecule detection using FISH-quant. (a) FISH-quant batch mode interface illustrating the quantifications for EXP1. The blue histograms represent the distribution of mRNA intensities (raw pixel data) plotted for all the mRNA detected for the ten stage positions, before (right) or after (left) filtering. The parameters can be further adjusted, by using the cursor changing the minimum and maximum intensities, to refine the mRNA detection. The narrow distribution of mRNA intensities, indicates that the detected spots represent a homogenous population of single mRNAs. (b) FISH-quant parameters for single mRNA averaging. FISH-quant main interface showing the parameters used for background estimation and signal to noise ratio (SNR) enhancement. (c) Average intensity of all single mRNAs processed. The first column is the maximum intensity projections along the three axes. The second column showsthe results of the fit with the Gaussian. The third column shows the residuals. (d) FISH-quant outline designer interface showing the cell outlines generated using Cellprofiler merged on the smFISH image.

Next all the smFISH images can be analysed using the same setting in the Batch processing mode that can be selected from the FISH-quant main interface. The setting and all the outlines are loaded and processed. The analysis for EXP1 is shown in Fig. 3a. The analysis file is saved using the Matlab format. This file was deposited (_FQ_batch_ANALYSIS_191205) and can be utilized to verify the mRNA detection parameters.

Following the batch processing, the intensity of single mRNAs is averaged (Fig. 3b–c) and it is used to set the parameters for measuring TS intensities (Fig. 3d). The file describing the averaged single mRNA intensity was deposited as well (_mRNA_AVG_ns). Once the settings tested for a single image are found, they are saved as a text file that can be used in the batch mode (Fig. 3a) to automatically detect TS intensities for all images. We deposited the file describing the nascent mRNA settings used to analyze EXP1 (_FQ__settings_NASCENT).

### 1.5 FISH-quant output results file

**Fig. 4:**
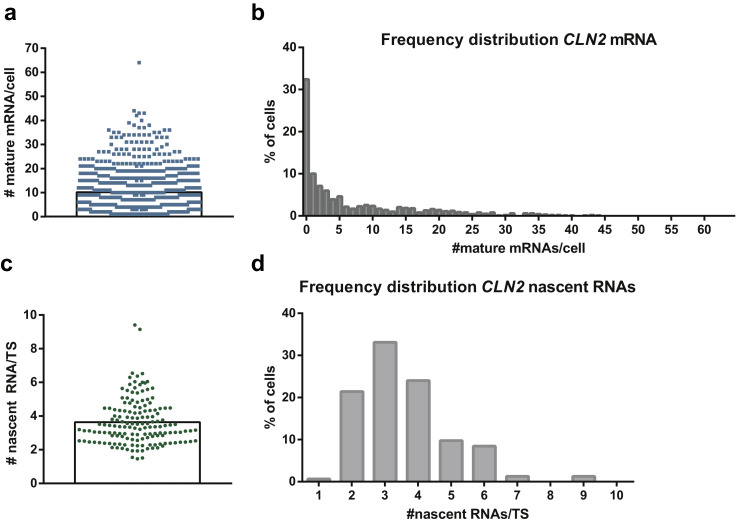
*CLN2* mature and nascent mRNA molecule quantification. (a) Quantification of *CLN2* mRNAs by smFISH in wild-type expressing cells, non-synchronized (*n* = 688). The histogram indicates the mean (mean ± 95% confidence interval for non-normally distributed data = 10.1 ± 0.7 mRNAs/cell). (b) Frequency distribution for *CLN2* mRNAs in wild-type expressing cells, non-synchronized (*n* = 1018). (c) Quantification of *CLN2* nascent RNAs by smFISH in wild-type expressing cells, non-synchronized (*n* = 154). The histogram indicates the mean (mean ± standard deviation = 3.6 ± 1.3 nascent RNA/TS). (d) Frequency distribution for *CLN2* nascent RNAs in wild-type expressing cells, non-synchronized (*n* = 154).

After the batch identification of both mRNAs and TS, several text files can be saved reporting the analysis output (e.g. the number of nascent and mature mRNAs per cell, the intensities and localization of the mRNAs). We deposited the text file reporting the number of nascent and mature *CLN2* mRNAs for the ten stage positions of EXP1 (__FQ_batch_summary_ALL_191204). This information can be further plotted to show, for instance, the number of mRNAs present in expressing cells (Fig. 4a), or the distribution of the mRNA in a population of cells (expressing and non-expressing) (Fig. 4b). Furthermore, the number of nascent mRNAs per transcription site can be plotted as individual values (Fig. 4c) or as a frequency distribution (Fig. 4d).

## 2 Experimental design, materials, and methods

### 2.1 Yeast cell cultures

The *S. cerevisiae* strain used in this article is BY4741 (MATa; his3Δ1; leu2Δ0; met15Δ0; ura3Δ0). Yeast cultures were exponentially grown in synthetic complete medium (SC-complete): Yeast nitrogen base without amino acids and with ammonium sulfate 6.7 g/L; SC powder with amino acids and supplements 2 g/L (Sunrise Technologies, 1300-030); Glucose 2% (w/v)). Cells were grown at 26 °C using constant shaking at 210 rpm.

### 2.2 Single molecule fish probes design

*CLN2* probes conjugated to Quasar 570 were designed using the Stellaris™ Probe Designer by LGC Biosearch Technologies and purchased from Biosearch Technologies (https://www.biosearchtech.com/support/tools/design-software/stellaris-probe-designer).

Probes sequences are the following:

**Table:** 

ttgatgacgagtcccatacg,	cggatagtagtccggtttag,	attctgcattagatagctca,	
ttcttgcagcatttcgaagt,	aacattggtggagatttctt,	gctggtctattagttttgga,	taatgttggaccttgtttcc,
ccacagacagctcgaacaaa,	ataccatttgtcactcgagt,	ctcttggaacaatagcggtc,	acaaccaatttggcttggtc,
agccaaccagagacaagtag,	atgatgtgattacaaccgcc,	ccagtagggatgactacatt,	gggttgggaccataaaatct,
cagagagtcgaggtatacgt,	gaccatcaccacagtaatga,	gtctagtatatgtctttcca,	gactgacgtttttcagagca,
tctacagtggcatcactatc,	tttaagtcttcttcttcttc,	ctaagtaagtcgtactgcca,	gagaatatgccgtgcgatac,
aaaggaccgtggtcttgatt,	gctttctgatgtcattggag,	atgccgttcattaaggtact,	cttccatcaaggagttagga,
agaacaccattgaccgtttt,	caagtgatattctttcact,	gttggatgcaatttgcagtt,	gatatggtaagctttctcga,
ttcgaaagagcatgatgggg,	gcgaaggaatggatgtgcta,	gagtgtggctttgagatgag,	atcagagagtgagctcatgt,
catattccggctgaaaacgc,	cttggagtgattggtgatga,	ctgctgaccaaattggtaca,	gtgctaccacatatactgtt,
ttcaccagactattcacact,	tttgttcgtagatcctttgt,	atcattggttgcgttattgc,	ttggttttccttgttagact,
attgaggtaatgcgccgttg,	ggggaacattccatggttaa,	ctatttatggtcccagttgg,	gatgaggcactgctagattt,
ggtattgcccataccaaaag			

### 2.3 Single molecule FISH combined with immunofluorescence

The detailed protocol to perform single molecule FISH combined with Immunofluorescence (smFISH-IF) is described in the accompanying article [1]. In brief, it was performed as follows. Yeast strains were grown overnight at 26 °C in SC-complete medium with 2% glucose. In the morning cells were diluted to OD_600_ 0.1 and allowed to grow until OD_600_ 0.3–0.4. Cells were fixed by adding paraformaldehyde (32% solution, EM grade; Electron Microscopy Science #15714) to a final concentration of 4% and gently shacked at room temperature for 45 min. Cells were then washed 3 times with buffer B (1.2 M sorbitol and 100 mM potassium phosphate buffer pH=7.5) and resuspended in 500 µL of spheroplast buffer (buffer B containing 20 mM VRC (Ribonucleoside–vanadyl complex NEB #S1402S), and 25 U of Lyticase enzyme (Sigma #L2524) per OD of cells for about 8 min at 30 °C. Digested cells were washed once with buffer B and resuspended in 1 mL of buffer B. 150 µL of cells were seeded on 18 mm (#1.5) poly-lysine treated coverslips and incubated at 4 °C for 30 min. Coverslips were washed once with buffer B, gently covered with ice-cold 70% ethanol and stored at −20 °C. For hybridization, coverslips were rehydrated by adding 2xSSC at room temperature twice for 5 min. Coverslips were pre-hybridized with a mix containing 10% formamide (ACROS organics #205821000)/2xSSC, at room temperature for 30 min. For each coverslip the probe mix (to obtain a final concentration in the hybridization mix of 125 nM) was added to 5 µL of 10 mg/µL E. coli tRNA/ ssDNA (1:1) mix and dried with a speed-vac. The dried mix was resuspended in 25 µL of hybridization mix (10% formamide, 2 × SSC, 1 mg/ml BSA, 10 mM VRC, 5 mM NaHPO4 pH 7.5) and heated at 95 °C for 3 min. Cells were then hybridized at 37 °C for 3 h in the dark. Upon hybridization coverslips were washed twice with the pre-hybridization mix for 30 min at 37 °C, once with 0.1% Triton X-100 in 2xSSC for 10 min at room temperature, once with 1×SSC for 10 min at room temperature.

Before the IF, the smFISH was fixed with 4% PFA- in PBS for 10 min at room temperature (RT). Coverslips were washed once with PBS 1× for 5 min at RT. The coverslips were pre-incubated with the IF solution (1×PBS, 0.1% RNAse-free Bovine Serum Albumin) for 30 min at RT. Next, the coverslips were incubated with the primary antibody (mouse anti-tubulin, 1:1000) in IF solution for 45 min at RT. The coverslips were washed with 1×PBS for 5 min at RT before incubation with the secondary antibody (goat anti-mouse Alexa 647, 1:1500, or goat anti-mouse Alexa 488, 1:1500) in PBS 1×, BSA 0.1%, for 45 min at RT. Excess antibody was removed by washing the coverslips three times with PBS1x for 5 min at RT. Before mounting, we dipped the coverslip in 100% ethanol and let them dry completely at RT, protected from the light. We mounted the coverslips face down onto a drop of ProLong Gold anti-fade mounting solution with DAPI placed on a glass slide. We allowed the mounting solution to polymerize at RT, overnight and in the dark.

To note, both secondary antibodies, conjugated with Alexa 647 or Alexa 488, can be used in combination with smFISH probes labelled with Quasar 570. Probes labelled with Quasar 670 can be combined with IF detected with secondary antibodies labelled with Alexa 555 or Alexa 488. Immunofluorescence can be also combined with two-color smFISH (protocols are provided in [4,11,12]).

### 2.4 Image acquisition

smFISH-IF data were collected by using a wide-field fluorescence microscope, combined with a CCD camera and a high numerical aperture objective corrected for chromatic aberration. Yeast cells attached on a glass coverslips were imaged at different wavelengths using the following conditions and order: Alexa 647 or Alexa 488 (IF): exposure 750 ms (100% lamp power); Quasar 570 (smFISH): 750 ms (100% lamp power); DAPI (nuclear staining): 50 ms (25% lamp power); DIC (cell outline): 150 ms. Ten to twenty different stage positions were acquired per experiment (to reach ~1000 cells for quantification). At each stage position, and for each fluorescent channel, we collected 41 Z-stacks every 200 nm. For DIC, one single Z-stack was acquired at the focal plane.

### 2.5 smFISH image analysis

smFISH images were analyzed using the Matlab written software FISH-quant [2]. The software is freely available, provided by Florian Mueller (https://research.pasteur.fr/en/member/florian-muller/) on the Bitbucket repository (https://bitbucket.org/muellerflorian/fish_quant/src/master/). The scripts are accompanied by an in depth-manual that explains the theory and how to perform single mRNA detection. The current version (V3) provides the scripts to perform automatic cell outline recognition using the freely available software Cellprofiler [13] as well as scripts to perform co-localization of spots in two-colors [14]. After background subtraction, the integrated fluorescence intensities of all detected cytoplasmic smFISH spots were fitted to a three-dimensional (3D) Gaussian to determine the average intensity and the coordinates of the mRNAs (Fig. 2). The peak intensity of the 3D Gaussian represents the intensity of a single *CLN2* mRNA hybridized with 48 single-labelled Quasar 570 probes. The average intensity of all the mRNAs was used to determine the number of transcripts at each transcription site (Fig. 3). smFISH performed with 48 20-nucleotide singly-labelled probes is likely to permit the detection of the majority of cellular mRNAs. This assumption is based on previous reports showing that two probe sets targeting different regions of the same mRNA and labelled with spectrally distinct fluorophores (e.g. Quasar 670 and Quasar 570) co-localize more than 80% of the time [4,12,15]. Furthermore, in an experiment performed in the model organism *Drosophila melanogaster*, it was shown that increasing the number of probes (e.g. from 48 to 63 or 91) did not significantly increase the number of mRNAs detected per cell by smFISH [16].

## Contributions

A.M. Investigation; Methodology; Data curation; Writing - review & editing. R.H.S. Funding acquisition; Supervision; Writing - review & editing. E.T. Conceptualization; Investigation; Methodology; Data curation; Funding acquisition; Supervision; Writing original draft; Writing - review & editing.
